# Pseudohyponatremia Leading to a Fatal Outcome in a Patient With Familial Hypertriglyceridemia

**DOI:** 10.7759/cureus.17066

**Published:** 2021-08-10

**Authors:** Amanda Dawson, Anish Kanukuntla, Priyaranjan Kata, Rana Ali, Pramil Cheriyath

**Affiliations:** 1 Internal Medicine, Hackensack Meridian Ocean Medical Center, Brick, USA; 2 Internal Medicine, Hackensack Meridian Health Jersey Shore University Medical Center, Neptune City, USA

**Keywords:** hyperlipidemia, hypertriglyceridemia, pseudohyponatremia, global cerebral edema, hyperproteinemia

## Abstract

Serum sodium assay is a commonly performed laboratory test in a clinical setting and the results are taken for granted without being aware of the actual methods involved. In conditions like hyperlipidemia and hyperproteinemia, excessive lipids in serum dilute the water component of the serum. Since sodium is dissolved only in the aqueous phase of serum, the sodium content per unit volume of plasma is reduced. Currently, most of the laboratories use the indirect ion-selective electrode method (ISE), where the plasma sample is diluted before the measurement. Indirect ISE may not give accurate results in conditions with higher serum lipid and protein levels. Overcorrection of the serum sodium levels in pseudohyponatremia may cause serious complications. We report a case of a 26-year-old Asian male with a past medical history of chronic pancreatitis, familial hypertriglyceridemia, and fatty liver who presented to the emergency department with acute pancreatitis. Initially, the patient was found to have hyponatremia and he was started on hypertonic saline for one day. Later the patient’s condition deteriorated and then it was determined that serum sodium results were a measurement artifact since the patient had extremely high levels of triglycerides. After realizing that it was a measurement artifact, the saline infusion was stopped and he was started on desmopressin. However, the patient deteriorated neurologically and expired later. As this patient had normal sodium levels, administration of hypertonic saline led to a fatal outcome.

## Introduction

Serum sodium assay is one of the most commonly performed laboratory tests in a hospital setting. Most often, the results are taken for granted without being aware of the actual methods involved in the lab procedures. However, in certain conditions, the reported lab results may not reflect the actual values, resulting in mismanagement of patients.

Hyponatremia is defined as the plasma sodium level less than 135 mmol/L and its prevalence in a hospital setting is up to 12% [1-3]. Pseudohyponatremia refers to low serum sodium in the presence of normal plasma tonicity [4-6]. In pseudohyponatremia, the water component is diluted by non-aqueous materials such as lipids and proteins. In conditions like hyperlipidemia and hyperproteinemia, abnormally high levels of these large molecules replace the aqueous phase of plasma, thus resulting in a reduced amount of serum electrolytes per unit volume of serum [6,7]. When measured with conventional flame photometry or indirect potentiometry, which involves prior dilution of the samples, the measured sodium concentration is low but the true plasma concentration is normal [7]. Not being aware of this artifact and correction of sodium in these conditions result in increased morbidity and mortality [8]. Since these concepts are not usually recognized in regular clinical practice, it would be helpful to review the methodology involved along with the events that led to a fatal outcome in this patient.

## Case presentation

**Figure 1:**
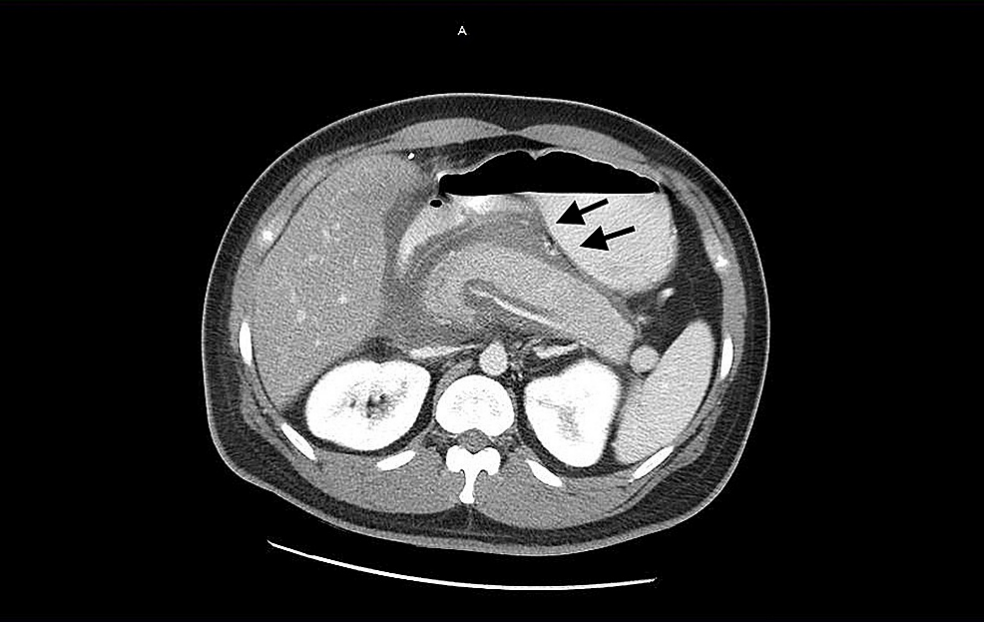
CT abdomen Imaging findings suggestive of severe acute pancreatitis with diffuse pancreatic enlargement, adjacent inflammatory changes, and free fluid.

A 26-year-old Asian male with a past medical history of chronic pancreatitis, familial hypertriglyceridemia, and fatty liver presented to the emergency department complaining of nausea and abdominal pain for one day that had gotten progressively worse. The pain was sharp in quality. At the time of presentation, his vital signs showed a temperature of 97 °F, a pulse of 107 beats per minute, a respiratory rate of 20 breaths per minute, and blood pressure of 188/99 mm Hg. Physical examination revealed extensive xanthomas located throughout his body. The abdomen was soft, mildly tender to palpation in the right upper quadrant, and bowel sounds were present. Further work up in the emergency room showed a WBC count of 12,000/mL (4.5-11.0 × 10^3^/µL), sodium 112 mEq/L (135-145 mEq/L), blood glucose 283 mg/dL (70-99 mg/dL), bicarbonate 18 mEq/L (23-30 mEq/L), anion gap 5 mEq/L (10-16 mEq/L), amylase 844 U/L (30-110 U/L), lipase 2170 U/L (10-140 U/L), and serum osmolality 331 mOsm/kg (275 to 295 mOsm/kg). The lipid profile showed elevated triglyceride levels of 5805 mg/dL (less than 150 mg/dL). CT scan of the abdomen and pelvis showed severe acute pancreatitis with diffuse pancreatic enlargement with inflammatory changes and free fluid (Figure 1).

**Figure 2:**
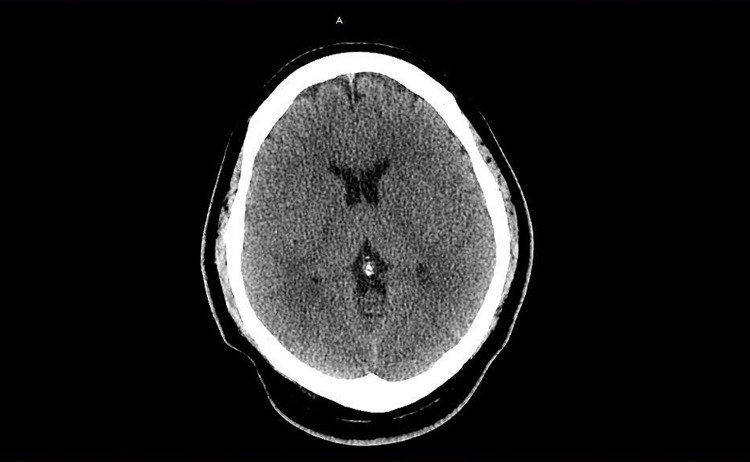
CT head Imaging findings suggestive of early global cerebral edema.

Later on that day, he was transferred to the ICU and requested to be seen by pulmonary critical care and nephrology. The patient was started on IV fluids. Because the patient's repeated lab values consistently revealed critically low sodium levels (109 meq/L), he was started on 3% saline at a rate of 25 ml/hour for the next eight hours with frequent electrolyte monitoring. Overnight, the patient was on hydromorphone for pain control. The following morning, the patient went into cardiac arrest; spontaneous circulation was restored in ten minutes. He was intubated and placed on ventilator support. His immediate arterial blood gas showed a PH of 7.09, PCO_2_ of 36.2, PO_2_ of 143.1, and a bicarbonate level of 10 meq/L. Later, it was realized that the patient had pseudohyponatremia due to his underlying hypertriglyceridemia. He was started on an IV bicarbonate drip and given one dose of desmopressin. Despite the ongoing treatment, the patient became unarousable and unresponsive to painful stimuli. On neurological examination, his eyes had an upward deviation with a sluggish pupillary response to light. An immediate metabolic panel was repeated which then showed a sodium level of 134 meq/L. A new CT of the head showed global cerebral edema (Figure 2). Because of the patient's poor prognosis, he was referred for comfort care after discussing with his family. Later that day, the patient passed away.

## Discussion

Human serum is composed of 93% water and 7% non-aqueous components (lipids, proteins, etc.) by volume [9]. All serum electrolytes including sodium are dissolved in the aqueous component. Increased serum lipid and protein components in blood result in a relative decrease in the water content of plasma but the proportions of serum electrolytes in the aqueous component remain unchanged. Serum sodium concentration is normally measured in milliequivalents per liter. In conditions like hyperlipidemia and hyperproteinemia, there is a relative decrease in plasma water content as it is replaced by serum lipids or proteins. In such cases, the measurement of serum sodium concentration is significantly reduced in the water component.

The measurement of sodium concentration on a larger scale in clinical laboratories became possible since the emergence of the flame emission spectrometer (FES). In this technique, the sample solution is diluted and a fine mist of the diluted aliquot is blown on a flame [10]. The intensity of the light emitted is directly proportional to the concentration of sodium in the sample [10]. But in current clinical practice, most of the laboratories use indirect potentiometry using an ion-selective electrode that has a sodium selective membrane, which measures the potential generated across a sodium selective membrane, when immersed in a serum sample [5,11]. The potential generated is a function of the activity of sodium ions in the sample [5,7,11]. The activity of an element is described as a measure of the number of atoms that act completely as ions per unit volume of electrolyte solution [12]. The activity and concentration of an element in a solution may be approximated when an electrolyte solution is at higher dilution [12]. Therefore, the plasma sample is diluted before it is being measured and corrected later for the degree of dilution based on the assumption that serum contains 7% solids by volume [1]. This indirect method has been proven to be appropriate under normal physiological conditions. However, in conditions like hyperlipidemia and hyperproteinemia, the fraction of solid-phase particles is increased substantially. When the same amount of diluent is added and with subsequent calculation of the sodium level based on the normal fraction of solid-phase particles results in an erroneously low plasma sodium level [1].

Our patient initially presented with features of acute pancreatitis with underlying hypertriglyceridemia. Initially, the patient was found to be having hyponatremia. As his labs showed severe hyponatremia, he was started on hypertonic saline for one day. Later the patient’s condition deteriorated and then it was determined that serum sodium results were a measurement artifact since the patient had extremely high levels of triglycerides. After realizing that it is a measurement artifact, the saline infusion was stopped and he was started on desmopressin. However, the patient deteriorated neurologically on the next day with abnormal findings on the CT scan. His overall clinical condition worsened which later resulted in death.

This extreme case demonstrates how normal sodium levels can masquerade as hyponatremia in a patient with hypertriglyceridemia which ultimately resulted in mismanagement, leading to a fatal outcome. Although the indirect ion-selective electrode method (ISE) for sodium measurement in serum has been widely used for its accuracy and validity under normal physiological conditions, the direct ISE method may be used in conditions with excessive levels of solid-phase components as this method gives a closer approximation to the actual sodium levels in such conditions. Unlike indirect ISE and flame photometry, direct ISE does not require prior dilution before measurement. In addition to using other methods, the water content of the plasma in patients with hyperlipidemia or hyperproteinaemia can be measured by the following formula [5]:

Plasma water content (percent) = 99.1 − (0.1 × L) − (0.07 × P)

where L and P refer to the total lipid and protein concentrations in g/L, respectively. With this formula, the sodium concentration can be adjusted to the normal value for plasma water content.

## Conclusions

Pseudohyponatremia is a measurement artifact when the fraction of solid-phase particles is more than the physiological range. In conditions like hyperlipidemia and hyperproteinemia, excessive lipids in serum dilute the water component of serum. Since sodium is dissolved only in the aqueous phase of serum, the sodium content per unit volume of plasma is reduced. Most of the laboratories use indirect ISE, where the plasma sample is diluted prior to the measurement. Since the total sodium content in the plasma is significantly reduced, the reported lab value is an underestimate. As the patient in fact has normal sodium levels, administration of hypertonic saline may lead to serious consequences, including death as in this patient. Clinicians must be cognizant about the methods involved in the measurement to prevent mismanagement in such conditions. Direct ISE may be an alternate option in these conditions.
